# Establishing the content validity of a new emergency department patient-reported experience measure (ED PREM): a Delphi study

**DOI:** 10.1186/s12873-022-00617-5

**Published:** 2022-04-09

**Authors:** Claudia Bull, Julia Crilly, Sharon Latimer, Brigid M. Gillespie

**Affiliations:** 1 School of Nursing and Midwifery, Menzies Health Institute Queensland, Griffith University, Southport, Queensland 4215 Australia; 2 Gold Coast Hospital and Health Service, Southport, Queensland 4215 Australia; 3 NHMRC Centre of Research Excellence in Wiser Wounds, Menzies Health Institute Queensland, Griffith University, Southport, Queensland 4215 Australia

**Keywords:** Patient-reported experience measures, Content validation, Validation studies, Consensus-building, Emergency department

## Abstract

**Background:**

Patient-reported experience measures aim to capture the patient’s perspective of what happened during a care encounter and how it happened. However, due to a lack of guidance to support patient-reported experience measure development and reporting, the content validity of many instruments is unclear and ambiguous. Thus, the aim of this study was to establish the content validity of a newly developed Emergency Department Patient-Reported Experience Measure (ED PREM).

**Methods:**

ED PREM items were developed based on the findings of a systematic mixed studies review, and qualitative interviews with Emergency Department patients that occurred during September and October, 2020. Individuals who participated in the qualitative interviews were approached again during August 2021 to participate in the ED PREM content validation study. The preliminary ED PREM comprised 37 items. A two-round modified, online Delphi study was undertaken where patient participants were asked to rate the clarity, relevance, and importance of ED PREM items on a 4-point content validity index scale. Each round lasted for two-weeks, with 1 week in between for analysis. Consensus was a priori defined as item-level content validity index scores of ≥0.80. A scale-level content validity index score was also calculated.

**Results:**

Fifteen patients participated in both rounds of the online Delphi study. At the completion of the study, two items were dropped and 13 were revised, resulting in a 35-item ED PREM. The scale-level content validity index score for the final 35-item instrument was 0.95.

**Conclusions:**

The newly developed ED PREM demonstrates good content validity and aligns strongly with the concept of Emergency Department patient experience as described in the literature. The ED PREM will next be administered in a larger study to establish its’ construct validity and reliability. There is an imperative for clear guidance on PREM content validation methodologies. Thus, this study may inform the efforts of other researchers undertaking PREM content validation.

**Supplementary Information:**

The online version contains supplementary material available at 10.1186/s12873-022-00617-5.

## Background

Patient-reported experience measures (PREMs) are instruments that capture the patient’s perspective of what happened during a care encounter, and how it happened [1]. PREMs differ to patient-reported outcome measures (PROMs), which are instruments used to measure a patient’s health and wellbeing (including physical and social functioning, psychological wellbeing, and symptom severity) [2, 3]. For more than 25 years, PREMs have been used to measure health systems performance and value-based healthcare internationally [4–10]. Value-based healthcare seeks to incentivise care providers and services for high quality care that supports improved patient outcomes, patient safety, clinical effectiveness and patient experiences [5, 7]. In the United States, 25% of annual hospital reimbursement via the Hospital Value-Based Purchasing Program is based on patient experience scores [11]. Similar schemes also operate in the United Kingdom in both primary and secondary care settings [12, 13]. In Australia, patient experience data is used to monitor health service quality and improvements [9], and establish key service performance indicators [14]. Thus, given the critical role that PREMs play in monitoring, evaluating and improving health services and systems globally, it is essential that they are valid and reliable instruments with strong conceptual foundations.

Despite the widespread use of PREMs, there are several challenges associated with measuring patient experiences. First, the concepts of patient experience and patient satisfaction are often used synonymously and interchangeably [15–17]. However, where patient experience captures an objective *report* of what happened during a care encounter and how it happened, patient satisfaction captures a subjective *evaluation* of the care experience; namely which of the patients’ expectations were met or not [16, 17]. Second, many PREMs exhibit varying levels of validity and reliability [1, 18–20]. Thus, there is some uncertainty regarding whether PREMs measure what they purport to measure (validity), and whether they are able to perform consistently (reliability) [21]. This calls into question the quality of the information many PREMs provide.

One aspect of validity that has been identified as missing or ambiguously reported for > 60% of PREMs is content validity [1]. Content validity is the extent that items of an instrument are relevant to representatives of the target population [22], and considers the importance, relevance and clarity of instrument items, domains and definitions; linguistics (e.g., terminology, grammar); how representative items are of the construct as a whole; and the adequacy and appropriateness of item response scales [22–24]. The COnsensus-based Standards for the selection of health Measurement INstruments (COSMIN) group notes that content validity is *“the most important measurement property of a patient-reported outcome measure (PROM).”* [23] Thus, it is arguably also the most important measurement property of a PREM.

The Delphi technique has emerged as a popular method for assessing instrument content validity [25, 26]. It seeks to obtain consensus on the opinion of experts through a series of structured survey rounds [27]. Yet, there is presently no published research on the use of the Delphi technique for PREM content validation. Thus, the aim of this study was to undertake a modified online Delphi study with patient participants to establish the content validity of a newly developed Emergency Department PREM (ED PREM).

## Methods

This study was guided by Delphi survey technique guidelines [27] and COSMIN guidance for content validation [23]. Ethical approval was received from Gold Coast Hospital and Health Services (Ref No: HREC/2020/QGC/61674) and Griffith University (Ref No: 2020/444). An online reactive Delphi technique was used, where experts ‘reacted’ to previously prepared information (e.g., survey items) as opposed to generating information in the first round [28]. In this study, experts (ED patients) were asked to:

1. Rate the relevance, importance and clarity of ED PREM items and response scales using a 4-point Content Validity Index (CVI) scale,
2. Suggest item and response scale revisions,
3. Suggest domain name and domain definition revisions, and
4. Suggest additional items for the ED PREM.

### Development of the ED PREM

ED PREM item generation consisted of two key steps: (i) domain identification, and (ii) item generation [29]. For domain identification, a systematic review was undertaken to understand whether there were valid and reliable instruments available in the peer-reviewed literature that capture patient experiences generally [1]. An existing review of ED PREMs was also consulted [18]. The results of both reviews demonstrated that existing instruments were limited by their length, ambiguous conceptual underpinnings, and heavy reliance on branch logic, which prevents existing PREM datasets from undergoing item reduction analysis such as exploratory factor analysis (as items tend to group where skip logic occurs, as opposed to where there are conceptual relations). Thus, a new ED PREM without such limitations was needed, with clear evidence of patient involvement in its development and content validation.

**Fig. 1:**
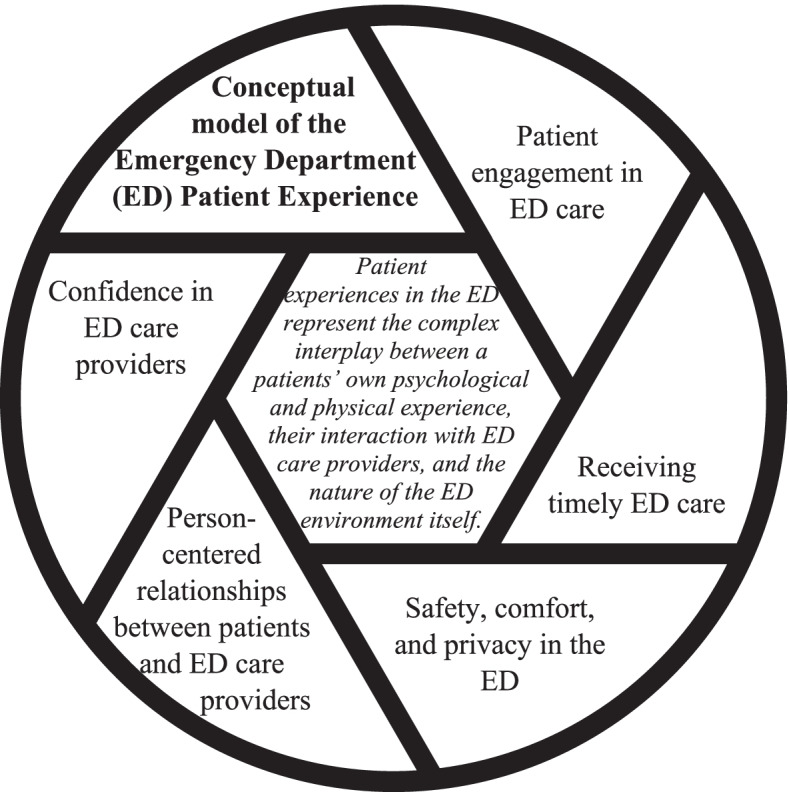
Conceptual model of Emergency Department (ED) Patient Experience

A systematic mixed studies review of patient experiences in the ED was subsequently undertaken, collating international evidence to gain a broad understanding of the key domains of patient experiences in the ED [30]. Additionally, qualitative interviews exploring patient experiences in the ED were undertaken (under review). There was substantive overlap in the findings of the review and qualitative studies. The systematic mixed studies review highlighted complex interplay between patients and their relationship with ED care providers and the ED environment [30]. The qualitative findings reinforced this notion, additionally emphasising the importance of specific relational attributes of care (i.e., person-centeredness, confidence, and engagement), as well as tangible and intangible ED environmental factors. These findings combined led to the development of a conceptual model of patient experiences in the ED (Fig. 1) and associated domain definitions (Table 1). This conceptual model guided the development of the initial list of ED PREM items.

**Table 1:** Conceptual model domain definitions

Conceptual model domain	Domain definition
1. Person-centred relationships between patients and ED care providers	Relationships between patients and care providers, founded on mutual respect, and an acknowledgement that the patient is a person (not a medical condition) with individual needs, values, and preferences.
2. Confidence in ED care providers	Patient perceptions of care providers’ knowledge, skill, and competence through the provision of thorough and comprehensive care.
3. Patient engagement in ED care	The opportunity patients have, and their capability, to be informed, involved, and included in their ED care to the extent they choose.
4. Safety, comfort, and privacy in the ED	Patient perceptions of safety, comfort (both physical and psychological), and privacy in the ED environment.
5. Receiving timely ED care	Patient perceptions of the timeliness in which they received ED care, and the extent to which they were informed about waiting and the progression of their ED care.

*ED* Emergency Department

The initial list of ED PREM items was reviewed and refined by the research team. Items were designed to: focus on a single aspect of the construct under investigation; be brief; have the potential to be interpreted the same way by all respondents; be understood by all respondents; and be grammatically simple where possible [29, 31, 32]. Item formatting, wording, and response options were taken into account [29, 32]. Flesh Reading Ease and Flesch-Kincaid Grade Level statistics were calculated to demonstrate the readability of ED PREM items. Reading Ease below 0.70 [33] and a Grade Level below 7 is considered appropriate [34]. This item list was subsequently employed in round one of the modified Delphi study.

### Expert panel recruitment

An expert was a patient who had recently received care in one of two EDs in Southeast Queensland, Australia. These experts, who had previously participated in a qualitative study with the research team, were purposively sampled for maximum variation of age, gender, and reason for presentation to the ED (under review). Thirty participants were interviewed relative to their availability to undertake a telephone interview within 2-weeks of their ED presentation. After being interviewed, participants were asked if they consented to being contacted in the future to participate in the Delphi study. Of the 30 patients interviewed, 24 (80%) consented to future participation. All potential experts were contacted via email or mobile, provided a brief overview of the study, and asked whether they were willing to participate. They were offered an AU$20 gift voucher to compensate for their time. Experts were eligible to participate in the Delphi study if they were aged 18 years or older; able to speak, read and comprehend English; and able to complete the Delphi survey independently online.

### Data collection

Round 1: Experts were sent an email invitation to participate in the round 1 survey in August 2021. After clicking on the survey link, participants were redirected to an online platform where they were asked to confirm their consent to participate, and rate each item and its’ response scale according to how clear, relevant, and important it was using a 4-point CVI scale where 1 = not clear/ relevant/ important, 2 = somewhat clear/ relevant/ important, 3 = quite clear/ relevant/ important, and 4 = highly clear/ relevant/ important [23]. This is the most frequently used variation of the CVI scale [35]. Using open dialogue boxes, experts were also asked to suggest item wording, domain name and domain definition revisions (if applicable); and suggest additional items for any experiential aspects of care missing. Demographic questions included gender, year of birth, highest educational qualification, identification as Aboriginal and/or Torres Strait Islander, and number of ED presentations in the past 12-months. Experts were given 2-weeks to complete the round 1 survey, after which time the survey was closed and results were exported into Microsoft Excel. A reminder email was sent to participants on days 5 and 12 of the round 1 survey period if they had not yet participated.

Round 2: The second round was a priori determined to be the final Delphi round, and commenced 1-week after the completion of round 1 in September 2021. Experts were emailed a second survey invitation and asked to rate the revised items relative to clarity, relevance, and importance using the 4-point CVI scale; and to suggest item revisions. Experts had 2-weeks to complete the round 2 survey, after which time the survey was closed and results were exported into Microsoft Excel. A reminder email was sent to participants on days 5 and 12 of the round 2 survey period if they had not yet participated.

### Data analysis

Round 1: Demographic and Delphi survey data were analysed descriptively using Microsoft Excel. Expert responses to item-level CVI (I-CVI) scales were binary coded as not or somewhat relevant/ important/ clear = 0, and quite or highly relevant/ important/ clear = 1. An I-CVI score was then calculated for each item as the number of experts scoring 1 relative to the total number of experts in the round 1 sample (proportion of agreement) [35]. Items that scored ≥0.80 for each of *relevance, importance* and *clarity* (without suggestions for revisions) were retained for the final ED PREM [36]. Items that scored ≥0.80 for each of *relevance, importance* and *clarity* (with suggestions for revisions), or ≥ 0.80 for each of *relevance* and *importance* but < 0.80 for *clarity* were revised by the research team based on expert feedback and included in the round 2 survey. Items that scored < 0.80 for each of *relevance, importance* and *clarity* were dropped from the ED PREM. Suggestions made by experts regarding changes to domain names, domain definitions, and missing items were also considered by the research team.

Round 2: Analysis of the round 2 survey results followed the same format as round 1. The research team scrutinised additional item revision suggestions before making further changes to the ED PREM. A scale-level CVI (S-CVI) score was also calculated as an average of I-CVI scores for all items included in the final ED PREM [35].

## Results

Table 2 depicts the demographic characteristics of the round 1 and 2 participants. Of the 18 individuals sent the round 1 survey, 15 participated in both round 1 (83.3%) and 2 (100%). The median age of the sample was 56 years (IQR 37-62.5), and two-thirds (66.7%) were female. The median number of presentations to the ED in past 12-months was 1 (IQR 1-2). Most participants were born in Australia (80.0%), and 6.7% identified as Aboriginal or Torres Strait Islander. One-third of participants had completed years 10-12 or equivalent secondary education, and an additional one-third held an Advanced Diploma/ Diploma.

**Table 2:** Demographic characteristics of round 1 and 2 participants

Demographic characteristics	Round 1 and 2 ***n*** = 15
	**Median (Q25-Q75)**
*Age (years)*	56 (37-62.5)
*Number of presentations in past 12-months*	1 (1-2)
	***n*** **(%)**
*Female*	10 (66.7%)
*Identified as Aboriginal and/ or Torres Strait Islander*	1 (6.7%)
*Country of birth*	
Australia	12 (80.0%)
New Zealand	2 (13.3%)
England	1 (6.7%)
*Highest level of completed education*	
Year 10-12 or equivalent (e.g., TAFE)	5 (33.3%)
Certificate III/IV	3 (20.0%)
Advanced Diploma/ Diploma	5 (33.3%)
Bachelor’s degree	1 (6.7%)
Postgraduate degree	1 (6.7%)

Q25 = 25th percentile; Q75 = 75th percentile; *TAFE* Technical and further Education

**Fig. 2:**
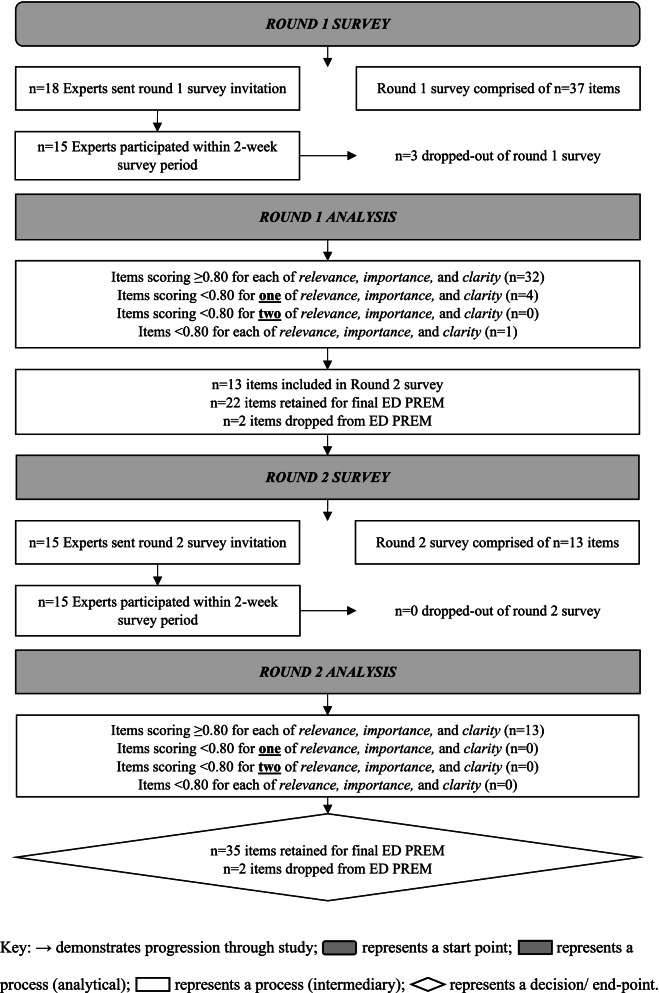
Flowchart of Delphi process, participants, and items

Figure 2 depicts the study process. The round 1 survey was comprised of 37 ED PREM items and had a Flesch Reading Ease score of 69.9, and a Flesch-Kincaid Grade Level of 5.5 (between grades 5 and 6). In round 1, 32 items scored ≥0.80 for each of clarity, relevance, and importance; 4 items scored ≥0.80 for two of clarity, relevance, and importance but < 0.80 for one of the criteria; and 1 item scored < 0.80 for all of clarity, relevance, and importance. Twenty-two items were retained for the final ED PREM after round 1; 2 items were dropped; and 13 items were revised and included in the round 2 survey. Question 1 in Domain two was dropped in round 1 despite I-CVI’s of 1.0 for each of clarity, relevance, and importance because several participants commented that it overlapped with question 2 of Domain 2. As such, these items were combined.

Of the 13 items included in the round 2 survey, all scored ≥0.80 for each of clarity, relevance, and importance. Thus, the resultant ED PREM comprised 35-items and had an S-CVI of 0.95. Table 3 shows the consensus decision and I-CVI scores for each item. Additional file 1 provides the final ED PREM.

**Table 3:** Item-level Content Validity Index (I-CVI) scores for each ED PREM item in Delphi survey rounds 1 and 2

Original ED PREM items	***Round 1***			Consensus decision	***Round 2***			Consensus decision
	Clarity I-CVI	Relevance I-CVI	Importance I-CVI		Clarity I-CVI	Relevance I-CVI	Importance I-CVI	
***Domain 1 – Person-centred relationships between patients and ED care providers***								
Q1: ED care providers were compassionate.	0.93	0.93	0.93	A	─	─	─	─
Q2: ED care providers were reassuring.	0.93	0.93	0.93	A	─	─	─	─
Q3: ED care providers listened to me.	0.93	1.00	1.00	A	─	─	─	─
Q4: ED care providers took me seriously.	0.93	0.93	1.00	A	─	─	─	─
Q5: ED care providers supported my decision to present to the ED.	1.00	0.87	0.80	A	─	─	─	─
Q6: ED care providers made me feel like I was no trouble to them.	1.00	1.00	1.00	A	─	─	─	─
Q7: ED care providers gave me the opportunity to talk.	0.87	1.00	1.00	A	─	─	─	─
Q8: ED care providers treated me like a person, not a medical condition.	1.00	1.00	1.00	A	─	─	─	─
Q9: ED care providers treated me with respect.	0.93	1.00	0.93	A	─	─	─	─
Q10: ED care providers were kind in how they treated me.	0.93	1.00	1.00	A	─	─	─	─
***Domain 2 – Confidence in ED care providers***								
Q1: ED care providers were competent at providing care.	1.00	1.00	1.00	D	n/a	n/a	n/a	n/a
Q2: ED care providers knew what they were doing.	1.00	0.93	0.93	R2	1.00	1.00	1.00	A
Q3: ED care providers were efficient.	0.93	0.87	0.80	R2	0.93	0.93	0.93	A
Q4: ED care providers were thorough.	0.87	1.00	1.00	R2	1.00	1.00	1.00	A
Q5: ED care providers worked well together.	0.87	0.87	0.87	A	─	─	─	─
Q6: ED care providers gave me consistent information.	0.87	1.00	1.00	R2	0.93	1.00	0.93	A
Q7: I was trusting of ED care providers.	0.87	1.00	1.00	A	─	─	─	─
Q8: I felt safe in the hands of ED care providers.	1.00	1.00	1.00	A	─	─	─	─
***Domain 3 – Patient engagement in ED care***								
Q1: ED care providers discussed my care with me.	0.87	0.93	0.93	A	─	─	─	─
Q2: ED care providers spoke to me in a way I could understand.	0.93	1.00	1.00	A	─	─	─	─
Q3: ED care providers encouraged me to ask questions.	0.80	0.93	0.87	A	─	─	─	─
Q4: ED care providers informed me of my care options.	0.73	0.93	0.93	R2	0.93	1.00	1.00	A
Q5: ED care providers involved me in decisions about my care as much as I wanted.	0.73	0.93	0.87	R2	0.80	1.00	1.00	A
Q6: ED care providers kept me informed throughout my ED journey.	0.93	0.93	0.93	A	─	─	─	─
***Domain 4 – Safety, comfort and privacy in the ED***								
Q1: I felt safe in the ED environment.	0.80	1.00	1.00	R2	0.87	1.00	1.00	A
Q2: I felt comfortable in the ED environment.	0.87	0.87	0.87	R2	0.87	1.00	1.00	A
Q3: I had access to the things I needed (e.g., toilets, wheelchairs, food and drinks).	1.00	1.00	1.00	A	─	─	─	─
Q4: The ED was clean.	1.00	1.00	0.93	A	─	─	─	─
Q5: The temperature in the ED was pleasant.	1.00	0.86	0.73	R2	0.93	0.80	0.80	A
Q6: The ED was quiet.	0.73	0.60	0.67	D	n/a	n/a	n/a	n/a
Q7: ED care providers discussed my personal details in a private manner.	0.87	0.93	1.00	A	─	─	─	─
Q8: ED care providers did all they could to make my space private.	0.87	0.87	0.93	R2	0.93	0.93	0.93	A
***Domain 5 – Receiving timely care***								
Q1: ED care providers informed me of how long I would be waiting to be seen.	0.87	0.80	0.80	R2	0.87	0.93	1.00	A
Q2: I was advised about why I needed to wait to receive care.	0.86	0.86	0.86	A	─	─	─	─
Q3: I received care in a prompt manner.	0.79	0.93	1.00	R2	1.00	1.00	1.00	A
Q4: ED care providers updated me throughout my ED journey about why I was waiting.	0.86	0.93	0.93	A	─	─	─	─
Q5: My ED journey progressed quickly.	0.86	0.93	0.93	R2	1.00	1.00	1.00	A
**S-CVI**								**0.95**

A = Accepted and retained for the final ED PREM; R2 = Revised and included in round 2; D = Item dropped from ED PREM; n/a = Not applicable; ─ = Not included in round; S-CVI = Scale-level content validity index

## Discussion

The purpose of this study was to reach consensus on the content of a new ED PREM. Patient experts assessed the 35-item ED PREM to have a high level of content validity, critically demonstrating that it captures experiential aspects of ED care that are meaningful to patients. The ED PREM will next be administered to a large-scale population where the ensuing responses will be used to evaluate additional aspects of its validity and reliability, and enable further item reduction. As there are few examples of PREM content validation in the peer-reviewed literature, this study can be used to inform other researchers in their own PREM content validation endeavours.

Two studies support the conceptual foundations of this ED PREM. First, a systematic mixed studies review, which described patient experiences in the ED as a complex interplay between patients, care providers and the ED environment [30]. Second, qualitative interviews with ED patients where patient experiences culminated into four themes; ‘Caring relationships between patients and ED care providers’, ‘Being in the ED environment’, ‘Variations in waiting for care’, and ‘Having a companion in the ED’ (under review). The findings from these two studies were combined to formulate the conceptual model of ED patient experience (Fig. 1) underpinning the development of the ED PREM. These conceptual foundations strongly align with existing literature, reinforcing the ED PREMs’ content validity, and suggesting its’ applicability to ED services broadly. Sonis and colleagues previously identified that the most commonly described themes of ED patient experience in the literature were staff-patient communication (described in 78% of included studies), ED wait times (56%), and staff empathy and compassion (44%) [37]. Australian research reported that patients place greatest value on the time they spend waiting, symptom relief, receiving a diagnosis and explanation of the problem, and friendly, caring and concerned ED staff [38, 39]. Additionally, a synthesis of qualitative research highlighted that emotions associated with an emergency situation (e.g., vulnerability and anxiety), staff-patient interactions, waiting, having family in the ED, and the emergency environment were characteristic of ED patient experiences [40]. Thus, not only does the newly developed ED PREM demonstrate good content validity from the patients’ perspective, but it also aligns with experiential aspects of ED care previously articulated in the literature.

The current study aimed to address a significant gap in the PREM development literature – the lack of PREM-specific guidance for content validation and psychometric evaluation methodologies more generally. A review of 88 PREMs identified that only 37.5% of instruments met COSMIN criteria for demonstrating appropriate content validation; content validation was either unclear or unknown for the others [1]. While COSMIN currently presents the best available criteria for good content validation processes [23], these criteria were developed for patient-reported outcome measures (PROMs) which are conceptually and operationally different to PREMs [2]. PROMs capture a patients’ health and wellbeing relative to care (e.g. physical functioning after surgery) [2]. The lack of PREM-specific guidance impacts on the standardisation and rigor of current practices used in PREM development. Thus, the development of PREM-specific content validation and psychometric evaluation guidance is an area of research that warrants investigation.

The use of the modified Delphi technique for this study presents several strengths relative to other consensus methodologies such as Nominal Group Technique (NGT) and Q-methodology. Briefly, NGT is conducted face-to-face and involves five highly structured steps that aim to facilitate effective group decision-making in response to a question [41–43]. Q-methodology involves participants ranking a set of items relative to a defined outcome (e.g., importance of those items), employing inverted factor analyses to interpret participant item rankings, and subsequently ascribing qualitative meaning to the resultant factor structure [44, 45]. The modified Delphi technique was advantageous because each round of the study was conducted anonymously and independently online. This gave each participant equal opportunity to have input into the study and reduced the risk of response bias that can arise in group settings (e.g., herd mentality or groupthink) [46]. The online capability also minimised the impact of COVID-19 on the conduct of the study. Additionally, each round took place over a two-week period, giving participants the flexibility to choose when and where they participated. This is not an option in NGT, where participants are required to attend a face-to-face meeting [43]. Finally, calculating I-CVIs and S-CVIs is analytically simple, whereas the analysis employed in Q-methodology requires a working knowledge of factor analysis [44]. Thus, this method may not be as feasible to those who are new to instrument development and psychometric evaluation.

A key consideration of this study was striking a balance between adequately representing the concept of ED patient experience, and ensuring that the number of items presented to patient participants was not overly burdensome. It has been suggested that for instrument development, *“the larger the item pool, the better”* [47]. Yet, while there is no prescribed optimal number of survey items, instruments that are shorter in length tend to have a higher response rate, and lower proportion of missing data when administered on a large-scale [48]. Thus, the resultant information is of greater quality and more likely to be generalisable to the target population. Most ED PREMs are over 40 items long, with response rates ranging between 18 and 51% depending on the mode of administration [18, 49, 50]. Thus, reducing respondent burden is critical to minimising the impacts of response biases and improving the quality of participant data [51]. Future psychometric evaluation of the ED PREM will further contribute to item reduction [52]. Thus, while items examined in content validation studies need to be comprehensive, minimising conceptually redundant items is also important for reducing participant burden both during content validation and subsequent administrations of the instrument.

### Limitations

A limitation of this study was that participants were only recruited from two EDs in Southeast Queensland. Additionally, females were over-represented, which does not reflect the reality that an equal distribution of women and men present to EDs in Australia [53]. Consequently, the ratings of clarity, relevance and importance for ED PREM items may not be representative of all Australian ED patient perspectives. However, the use of a maximum variation sampling frame aimed to minimise this by ensuring that individuals with wide-ranging demographic and clinical characteristics were involved in the study.

## Conclusions

As patient experiences become increasingly integral to measuring value in healthcare across services and systems internationally, it is critical that the experiential attributes of healthcare captured by PREMs are meaningful to patients. Thus, examining PREM content validation in the eyes of patients is critical. We used a modified, online Delphi technique to demonstrate the content validity of a 35-item ED PREM that will now undergo further psychometric evaluation. This study can be used to inform content validation methods and procedures of other PREMs, and supports the need for PREM-specific guidance on content validation and psychometric evaluation more generally.

## Supplementary Information

**Additional file 1. **Final ED PREM. Supplementary file providing the final version of the ED PREM (including full items and response options).

## Data Availability

All data generated or analysed during this study are included in this published article.

## Abbreviations

COSMIN: COnsensus-based Standards for the selection of health Measurement INstruments
CVI: Content Validity Index
ED: Emergency Department
ED PREM: Emergency Department Patient-Reported Experience Measure
HREC: Human Research Ethics Committee
I-CVI: Item-level Content Validity Index
IQR: Interquartile Range
NGT: Nominal Group Technique
PREM: Patient-Reported Experience Measure
PROM: Patient-Reported Outcome Measure
S-CVI: Scale-level Content Validity Index
