# Supplementary material for: Establishing the content validity of a new emergency department patient-reported experience measure (ED PREM): a Delphi study
Source: BMC Emerg Med. 2022 Apr 9;22:65. doi: 10.1186/s12873-022-00617-5 (PMC8994175; doi:10.1186/s12873-022-00617-5)
Supplement: Supplementary file 1 — Additional file 1. Final ED PREM. Supplementary file providing the final version of the ED PREM (including full items and response options). [file 12873_2022_617_MOESM1_ESM.docx]

**Supplementary material 1: Final ED PREM**

| **Item *n*** | **Item** | **Response categories** | | | | |
| --- | --- | --- | --- | --- | --- | --- |
| ***Person-centred relationships between patients and ED care providers*** | | | | | | |
| 1 | ED care providers were compassionate. | Never | Rarely | Sometimes | Very often | Always |
| 2 | ED care providers were reassuring. | Never | Rarely | Sometimes | Very often | Always |
| 3 | ED care providers listened to me. | Never | Rarely | Sometimes | Very often | Always |
| 4 | ED care providers took me seriously. | Never | Rarely | Sometimes | Very often | Always |
| 5 | ED care providers supported my decision to present to the ED. | Never | Rarely | Sometimes | Very often | Always |
| 6 | ED care providers made me feel like I was no trouble to them. | Never | Rarely | Sometimes | Very often | Always |
| 7 | ED care providers gave me the opportunity to talk. | Never | Rarely | Sometimes | Very often | Always |
| 8 | ED care providers treated me like a person, not a medical condition. | Never | Rarely | Sometimes | Very often | Always |
| 9 | ED care providers treated me with respect. | Never | Rarely | Sometimes | Very often | Always |
| 10 | ED care providers were kind in how they treated me. | Never | Rarely | Sometimes | Very often | Always |
| ***Patient confidence in ED care providers*** | | | | | | |
| 1 | ED care providers were experienced and knew what they were doing. | Never | Rarely | Sometimes | Very often | Always |
| 2 | ED care providers were time efficient in how they cared for me. | Never | Rarely | Sometimes | Very often | Always |
| 3 | ED care providers were thorough in how they cared for me. | Never | Rarely | Sometimes | Very often | Always |
| 4 | ED care providers gave me consistent information throughout my ED journey. | Never | Rarely | Sometimes | Very often | Always |
| 5 | ED care providers worked well together. | Never | Rarely | Sometimes | Very often | Always |
| 6 | I was trusting of ED care providers. | Never | Rarely | Sometimes | Very often | Always |
| 7 | I felt safe in the hands of ED care providers. | Never | Rarely | Sometimes | Very often | Always |
| ***Patient engagement in ED care*** | | | | | | |
| 1 | ED care providers discussed my care with me. | Never | Rarely | Sometimes | Very often | Always |
| 2 | ED care providers spoke to me in a way I could understand. | Never | Rarely | Sometimes | Very often | Always |
| 3 | ED care providers encouraged me to ask questions. | Never | Rarely | Sometimes | Very often | Always |
| 4 | ED care providers informed me of my treatment options. | Never | Rarely | Sometimes | Very often | Always |
| 5 | ED care providers involved me in decisions about my treatment as much as I wanted. | Never | Rarely | Sometimes | Very often | Always |
| 6 | ED care providers kept me informed throughout my ED journey. | Never | Rarely | Sometimes | Very often | Always |
| ***Safety, comfort, and privacy in the ED*** | | | | | | |
| 1 | I felt physically safe in the ED environment. | Never | Rarely | Sometimes | Very often | Always |
| 2 | I felt comfortable in the ED environment (both physically and emotionally). | Never | Rarely | Sometimes | Very often | Always |
| 3 | I had access to the things I needed (e.g., toilets, wheelchairs, food and drinks). | Never | Rarely | Sometimes | Very often | Always |
| 4 | The ED was clean. | Never | Rarely | Sometimes | Very often | Always |
| 5 | The temperature in the ED was comfortable. | Never | Rarely | Sometimes | Very often | Always |
| 6 | ED care providers discussed my personal details in a private manner. | Never | Rarely | Sometimes | Very often | Always |
| 7 | ED care providers did all they could to make my treatment space private. | Never | Rarely | Sometimes | Very often | Always |
| ***Receiving timely care*** | | | | | | |
| 1 | I was informed about how long I might have to wait when I first arrived to the ED. | Never | Rarely | Sometimes | Very often | Always |
| 2 | I was advised about why I needed to wait to receive care. | Never | Rarely | Sometimes | Very often | Always |
| 3 | I received care in good time considering the nature of my condition. | Never | Rarely | Sometimes | Very often | Always |
| 4 | ED care providers updated me throughout my ED journey about why I was waiting. | Never | Rarely | Sometimes | Very often | Always |
| 5 | My ED journey progressed in good time considering the nature of my condition. | Never | Rarely | Sometimes | Very often | Always |

ED = Emergency Department
